# Supplementary material for: Signatures of sliding Wigner crystals in bilayer graphene at zero and finite magnetic fields
Source: Nat Commun. 2025 Oct 7;16:8921. doi: 10.1038/s41467-025-64587-5 (PMC12504728; doi:10.1038/s41467-025-64587-5)
Supplement: Supplementary file 1 — Supplementary Information [file 41467_2025_64587_MOESM1_ESM.pdf]

Supplementary Information

for

"Signatures of sliding Wigner crystals in bilayer graphene

at zero and finite magnetic fields"

Anna M. Seiler[1,*], Martin Statz[1], Christian Eckel[1], Isabell Weimer[1], Jonas Pöhls[1], Kenji Watanabe[2], Takashi Taniguchi[3], Fan Zhang[4], R. Thomas Weitz[1,*]

[1]1st Physical Institute, Faculty of Physics, University of Göttingen, Friedrich-Hund-Platz 1, 37077 Göttingen, Germany

[2]Research Center for Functional Materials, National Institute for Materials Science, 1-1 Namiki, Tsukuba 305-0044, Japan

[3]International Center for Materials Nanoarchitectonics, National Institute for Materials Science, Tsukuba, Japan

[4]Department of Physics, University of Texas at Dallas, Richardson, TX, 75080, USA

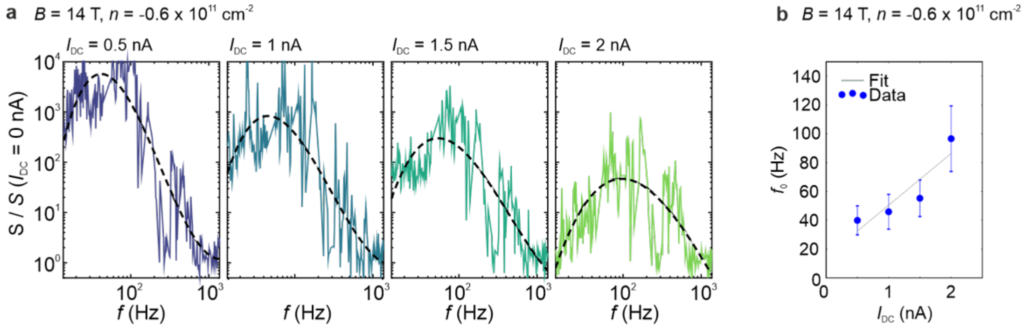

Supplementary Fig. 1. Frequency dependence of the spectral noise density within the magnetic-field induced Wigner crystal state. (a) Spectral noise density S normalized with respect to S (I_{DC} = 0 nA) as a function of the applied AC frequency f (I_{AC} = 100 pA) measured at different dc currents I_{DC}, charge carrier density n = -0.6 x 10^{11} cm^{-2}, out-of-plane magnetic field $B_{\perp}$ = 14 T and electric displacement field D = 0.15 Vnm^{-1}. Data points that correspond to the noise floor of the measurement setup and the 50 Hz electrical grid were removed (see Methods). The noise bulges are fitted with polynomial functions represented by dashes lines. Note that these measurements were conducted one year after those presented in Fig. 1 in the manuscript. While the overall noise level has changed, the frequency dependence remains consistent. **(b)** Dependence of the washboard frequency f_0 extracted from **(a)**. Error bars represent the uncertainty in the extracted peak frequency from the polynomial fit. A linear fit is shown in grey.

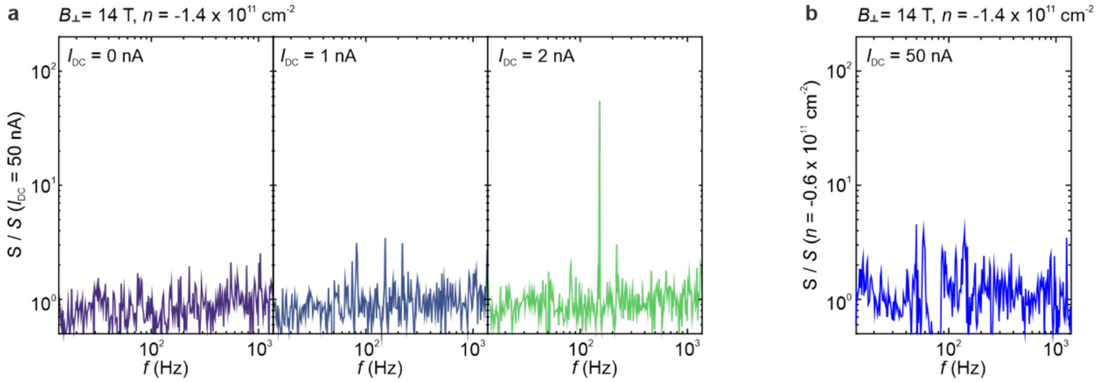

Supplementary Fig. 2. Frequency dependence of the spectral noise density within a fractional quantum Hall state. (a) Normalized spectral noise density S relative to the background spectral noise density measured at I_{DC} = 50 nA as a function of the applied frequency f of the AC current I_{AC} at different I_{DC} at an out-of-plane magnetic field B= 14 T, an electric displacement field D = 0.15 Vnm^{-1} and a charge carrier density of n = -1.4 x 10^{11} cm^{-2} (fractional QHS with filling factors ν = 1/3). No features in S can be discerned. **(b)** S relative to the background spectral noise density measured at n = -0.6 x 10^{11} cm^{-2} (see Fig. 1 for more data taken at n = -0.6 x 10^{11} cm^{-2}) as a function of f.

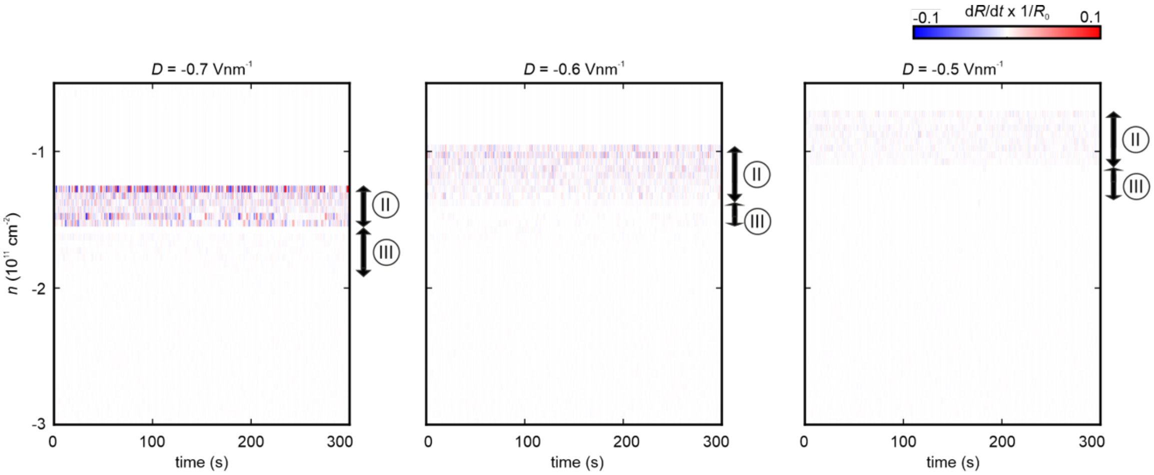

Supplementary Fig. 3. Noise at different electric displacement fields. Derivative of the normalized resistance over time $\frac{dR}{dt} \times \frac{1}{R_0}$ as a function of the charge carrier density *n* at different electric displacement field *D*, with an applied AC bias current of 1 nA and without applied DC bias current. Fluctuations in the resistance become less pronounced with decreasing $|D|$ and move towards smaller *n*, following the phase boundaries.

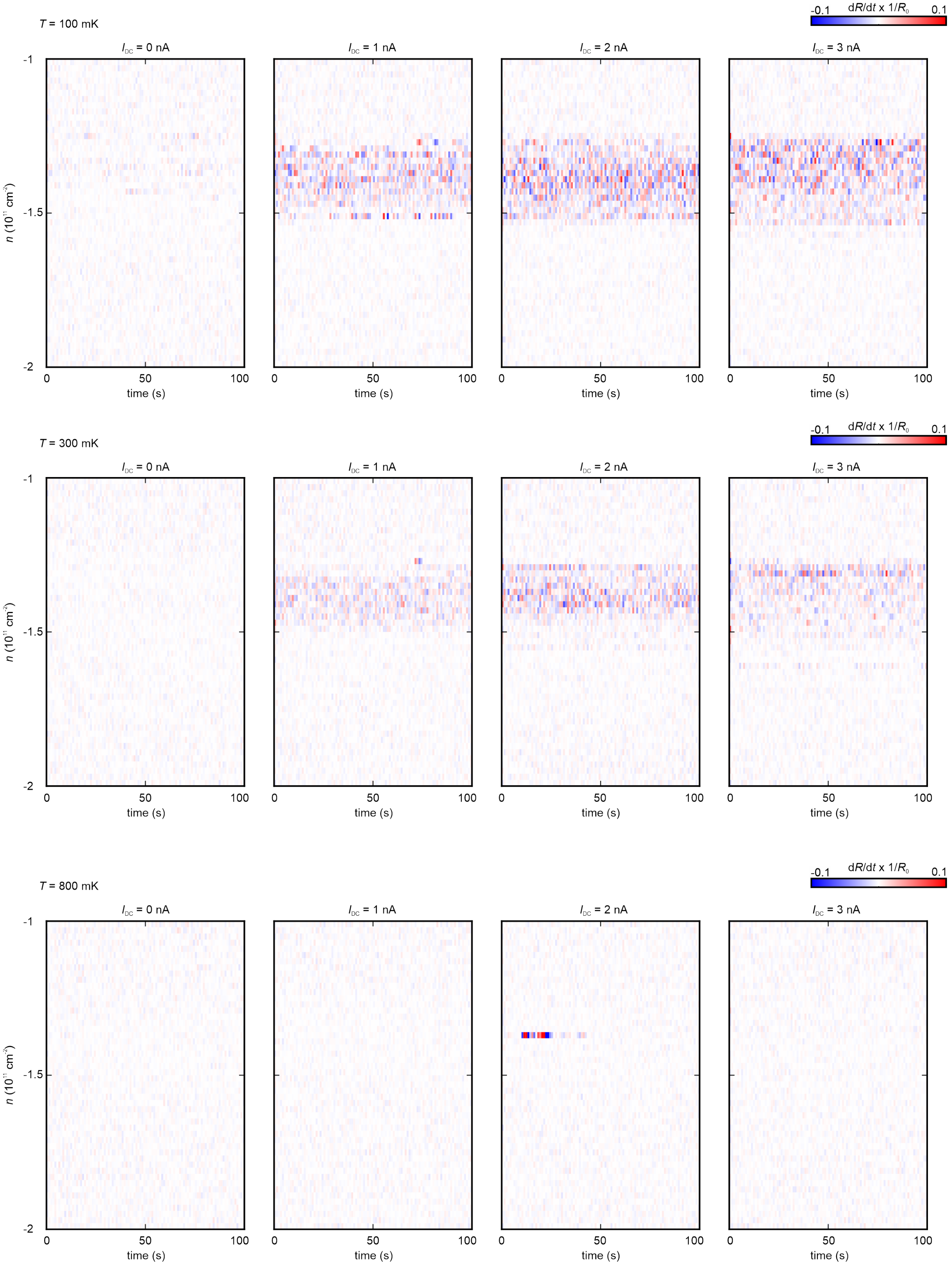
T = 100 mK
-0.1
dR/dt x 1/R0
0.1
IDC = 0 nA
IDC = 1 nA
IDC = 2 nA
IDC = 3 nA
n (10^11 cm^-2)
-1
-1.5
-2
0
50
100
time (s)
T = 300 mK
T = 800 mK

Supplementary Fig. 4 Noise as a function of temperature. Derivative of the normalized resistance over time dR/dt x 1/R_0 as a function of the charge carrier density *n* at different DC bias currents and different temperatures *T.* An AC bias current of 100 pA was applied at a frequency of 78 Hz. The electric displacement field *D* was set to -0.7 Vnm^{-1}. Fluctuations in the resistance become less pronounced with increasing *T*.

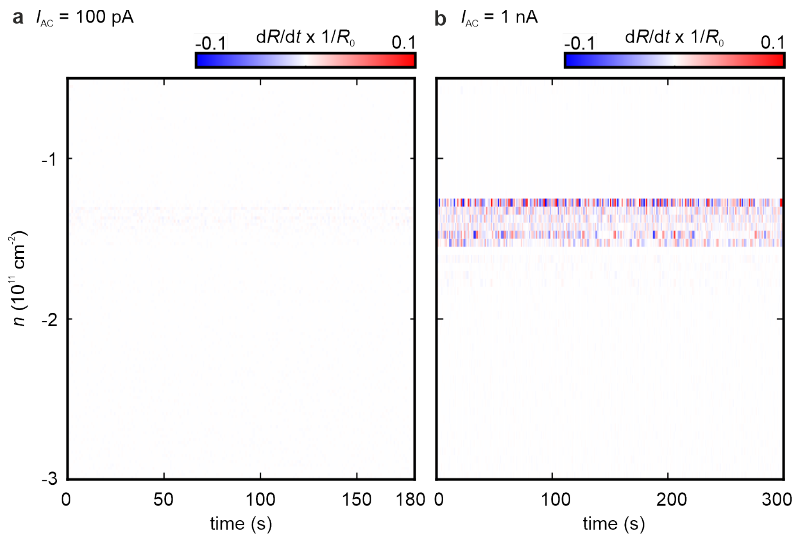

Supplementary Fig. 5. Noise as a function of the AC currents. Derivative of the normalized resistance over time dR/dt x 1/R_0 as a function of the charge carrier density *n* and an applied AC bias current of 100 pA **(a)** and 1 nA **(b)** at an electric displacement field of -0.7 Vnm^{-1} and a frequency of 78 Hz. No DC bias current was applied.

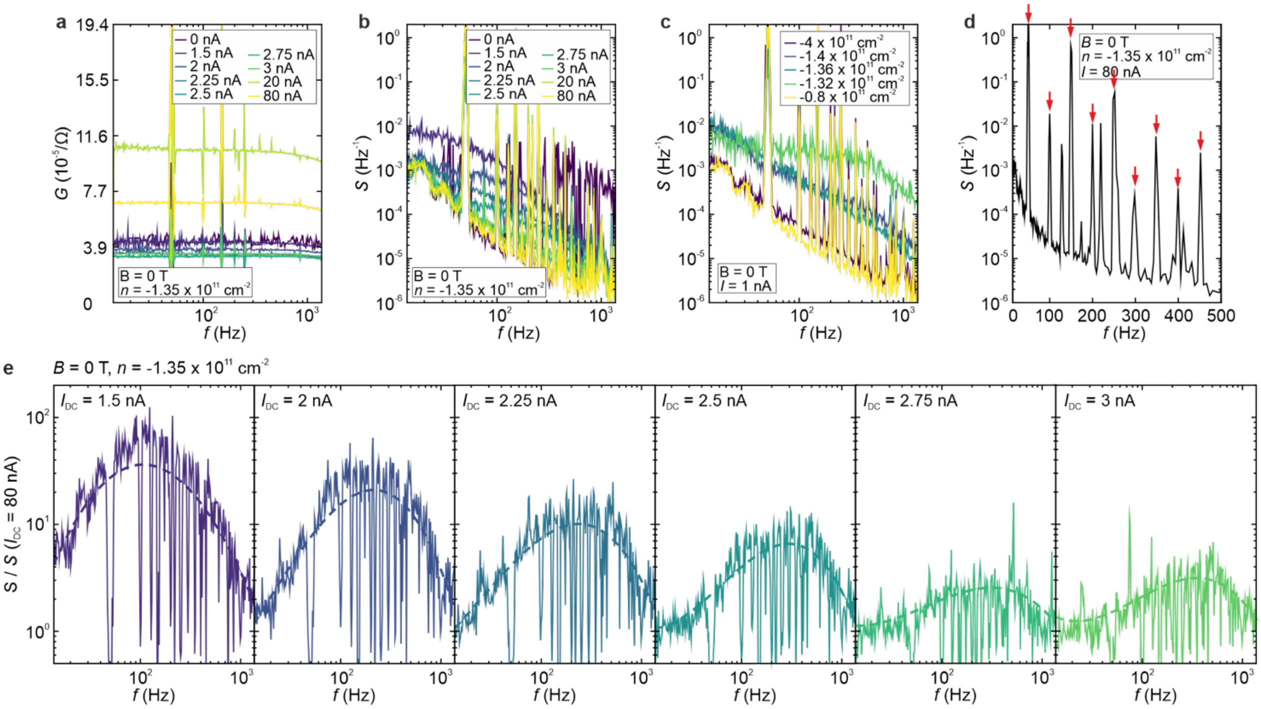

Supplementary Fig. 6. Frequency dependence of the conductance and spectral noise density. Conductance *G* **(a)** and normalized spectral noise density *S* **(b)** as a function of the applied AC frequency *f* (I_{AC} = 100 pA) measured at different DC currents I_{DC} at zero magnetic field *B*, charge carrier density *n* = -1.35 x 10^{11} cm^{-2} and electric displacement field *D* = -0.7 Vnm^{-1}. *G* is nearly independent of *f* for f < 1 kHz and decreases for *f* > 1 kHz due to electrical filters integrated into the cryostat. *G* peaks around 50 Hz and its higher harmonics arise from the electrical grid. **(c)** *S* as a function of *f* at I_{AC} = 100 pA, I_{DC} = 1 nA and at different *n*. S increases in the density regime of phase II (*n* = -1.32 x 10^{11} cm^{-2}, *n* = -1.36 x 10^{11} cm^{-2} and *n* = -1.4 x 10^{11} cm^{-2}) but is independent of *n* outside of phase II. **(d)** *S* measured as a function of *f* at *n* = -1.35 x 10^{11} cm^{-2}, *B* = 0 T and *I* = 80 nA, plotted on a linear scale in *f*. Peaks associated with 50 Hz noise, corresponding to 50 Hz electrical grid, are highlighted by red arrows. These data points were removed in the Fig. 5. **(e)** *S* / *S* (I_{DC} = 80 nA)) measured as a function of *f* at different I_{DC} before removing data points that correspond to the noise floor of the measurement setup and the 50 Hz electrical grid.

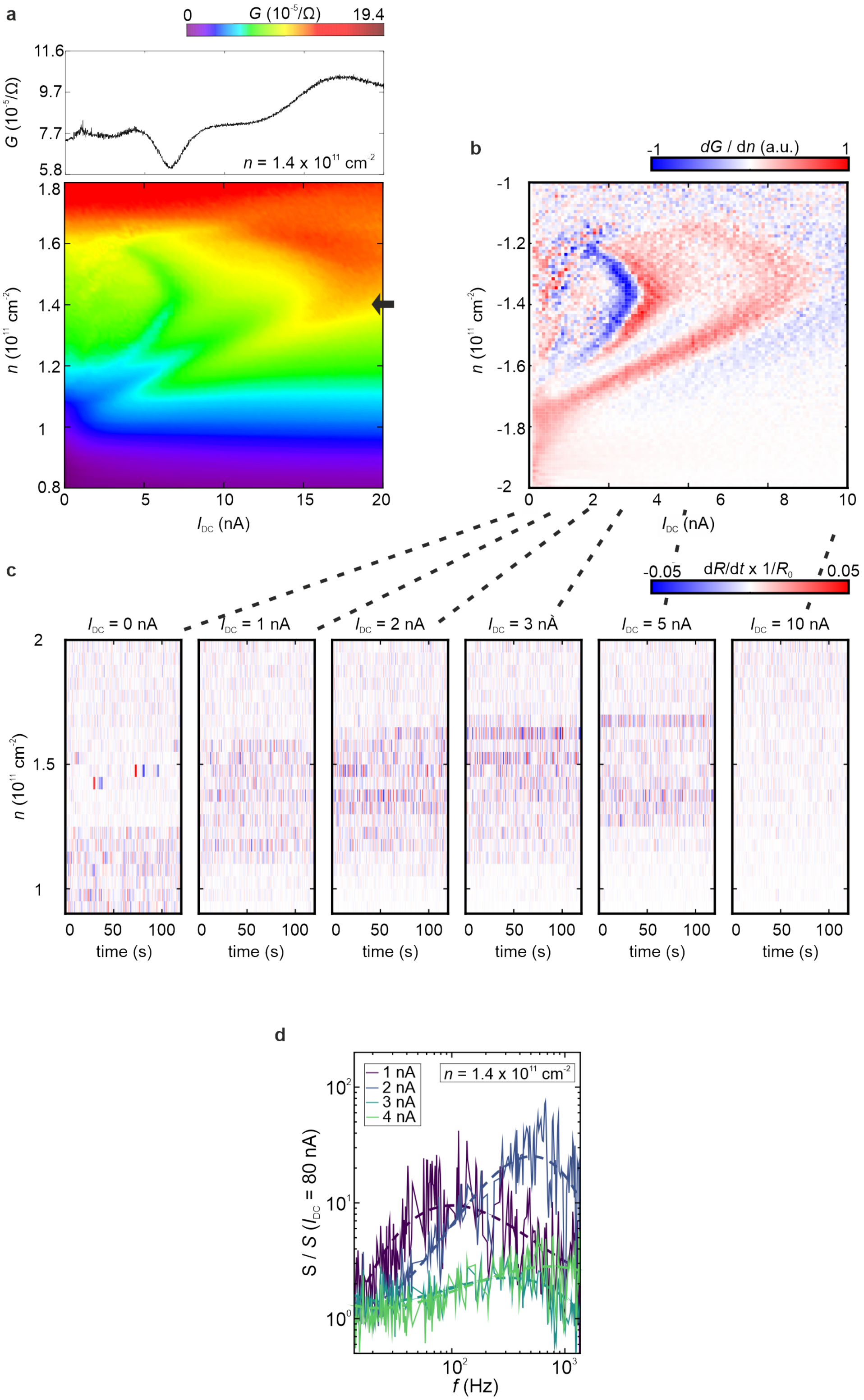

Supplementary Fig. 7. Noise in the spin and valley-polarized insulating (svi) phase at electron-doping. (a,b) Conductance (G) **(a)** and derivative of the conductance (dG/dI) **(b)** as a function of the applied DC current I_{DC} and the charge carrier density n at an electric displacement field of D = -0.8 Vnm^{-1} and in the

density regime of the spin and valley-polarized insulating (svi) phase (see Reference [24]). A linecut taken at n = 1.4 x 10^{11} cm^{-2} is shown in the top. **(c)** Derivative of the normalized resistance over time dR/dt x $1/R_0$ as a function of time t and n at different I_{DC}. Noise, indicative of depinning of the Wigner crystal or Wigner solid state, is present in the density regime of the svi phase at I_{DC} ranging from 1 nA to 5 nA. Additionally, noise is present at I_{DC} = 0 and small n due to the extremely high resistances within this regime that cannot be resolved by our measurement setup. **(d)** Spectral noise density S normalized with respect to S (I_{DC} = 80 nA) as a function of the applied AC frequency f (I_{AC} = 100 pA) and measured at different I_{DC} and n =-1.4 x 10^{11} cm^{-2}. Frequency-dependent noise bulges appear for currents ranging from 1 nA to 4 nA. Data points that correspond to the noise floor of the measurement setup and the 50 Hz electrical grid were removed (see Methods).

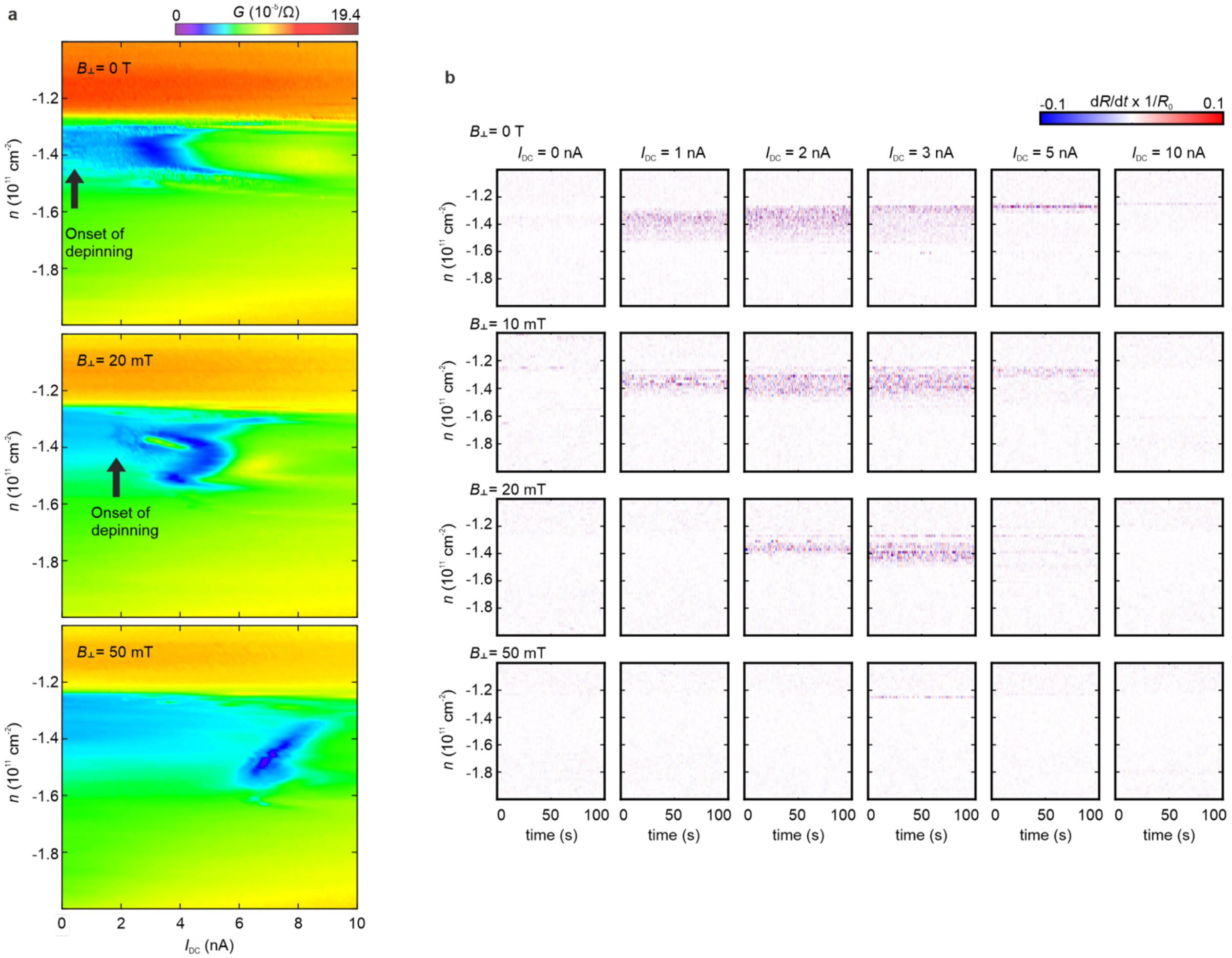

Supplementary Fig. 8. Noise as a function of the out-of-plane magnetic field. (a) Conductance (*G*) with as a function of the applied DC current I_{DC} and the charge carrier density *n* at different out-of-plane magnetic fields $B_\perp$. The onset of depinning moves to higher values of I_{DC} with increasing $B_\perp$. **(b)** Derivative of the normalized resistance over time dR/dt x 1/R_0 as a function of time *t* and *n* at different I_{DC} and $B_\perp$. No noise, indicative of depinning of the Wigner crystal or Wigner solid state, is present at $B_\perp$ = 50 mT.
